# Gastrointestinal Manifestation of COVID-19 in Hospitalized Patients

**DOI:** 10.7759/cureus.18024

**Published:** 2021-09-16

**Authors:** Naintara Sukharani, Jitesh Kumar, Kainat Makheja, Pinky Bai, Sucheta Sharma, Komal Subash, Yasir Kamawal, Simra Shahid, Amber Rizwan

**Affiliations:** 1 Internal Medicine, Peoples University of Medical and Health Sciences For Women, Karachi, PAK; 2 Internal Medicine, Ghulam Muhammad Mahar Medical College, Sukkur, PAK; 3 Internal Medicine, Jinnah Sindh Medical University, Karachi, PAK; 4 Internal Medicine, Punjab Institute of Medical Sciences, Jalandhar, IND; 5 Internal Medicine, Baqai Medical University, Karachi, PAK; 6 Family Medicine, Jinnah Postgraduate Medical Center, Karachi, PAK

**Keywords:** sars-cov-2 (severe acute respiratory syndrome coronavirus -2), nausea, covid-1, vomiting, diarrhea, gastrointestinal symptoms

## Abstract

Introduction: During the initial phase of the pandemic, gastrointestinal (GI) symptoms were less frequent but during the later stages, GI manifestations have become more frequent. This study aims to explore the prevalence of GI symptoms in COVID-19 patients, and also focuses on the frequency of these symptoms.

Methods: This longitudinal study was conducted in a COVID-19 unit of a tertiary care hospital, Pakistan. Data of patients hospitalized with COVID-19 infection between June 2021 and July 2021 was included in the study. A total of 412 participants were enrolled in the study via consecutive convenient non-probability sampling. Participants’ symptoms and demographics were noted in a self-structured questionnaire. The collected data were analyzed using Statistical Package for Social Sciences (SPSS), version 23.0 (IBM Corp., Armonk, NY).

Results: A total of 261 (63.3%) participants had a minimum of one GI symptom. The most common symptom was anorexia (43.9%), followed by diarrhea (24.7%) and nausea/vomiting (17.9%).

Conclusion: Our study indicates high frequency of COVID-19 patients reporting GI symptoms. Anorexia, diarrhea, nausea, and vomiting were commonly reported symptoms. Therefore, COVID 19 testing should be considered with patients presenting with GI symptoms.

## Introduction

Coronavirus disease-2019 (COVID-19), also known as severe acute respiratory syndrome coronavirus 2 (SARS-CoV-2), was initially reported in December 2019 in the city of Wuhan, China. Since then, it has spread rapidly throughout the majority of the countries and has consequently become the greatest public health challenge of its time [1].

It is zoonotic in origin and is the third documented coronavirus of animal origin that has infected humans in the last 20 years [2]. As of May 2020, the World Health Organization reported approximately 4,995,996 confirmed cases and 327,821 deaths secondary to SARS-CoV-2 infection [3]. It is transmitted via the respiratory droplets from an infected individual either due to the inhalation of the droplets or via direct contact. The infected individual may be presymptomatic, asymptomatic or a symptomatic carrier [4]. The incubation period ranges from 2-14 days whilst the average time from exposure to the virus to the onset of symptoms is five days, with the majority of the people developing symptoms within 11.5 days. Its rate of spread is very high and is considered to be greater than the rate of spread for SARS-CoV-1 and the Middle East respiratory coronavirus [3,4].

It can manifest as asymptomatic to fulminant disease which can result in acute respiratory failure as well as sepsis [4]. COVID-19 infection manifests with a variety of symptoms, the most common of which includes fever with or without chills, dry cough, shortness of breath, myalgia, headache, a loss of taste or smell, sore throat, runny nose, nausea, vomiting, and diarrhea. Some of the emergency symptoms include troubled breathing, constant chest pain, cyanosis, and sudden confusion [5]. Fever is known to be the most frequent symptom, followed by cough, dyspnea, and fatigue. However, the less common symptoms are sputum production, headache, hemoptysis, and diarrhea [6].

During the initial phase of the pandemic, the gastrointestinal (GI) symptoms were less frequent but during the later stages, [6] the GI manifestations have become more frequent. COVID-19 can manifest digestive symptoms in the absence of respiratory symptoms [7]. The GI manifestations, including anorexia, diarrhea, vomiting, nausea, abdominal pain, and GI bleeding are reported, with diarrhea being the commonest symptom in both adults and children [7]. In a study, six patients underwent endoscopy, upon which esophageal bleeding with erosions and ulcers was reported in one patient and two patients had COVID-19 RNA in esophageal, gastric, duodenal, and rectal samples. This may indicate that the COVID-19 infection can target the GI tract and use it as a mode of transmission [8,9]. The digestive symptoms are known to increase the risk for acute respiratory distress syndrome [10].

Although there is a lot of evidence on the association between GI manifestations and COVID-19, but with the mutations in the virus, the effects on the frequency of the symptoms and better treatment options for such patients need to be focused on. This study aims to explore the prevalence of GI symptoms in COVID-19 patients, and also focuses on the frequency of these symptoms.

## Materials and methods

This longitudinal study was conducted in a COVID-19 unit of a tertiary care hospital, Pakistan. Data of patients hospitalized with COVID-19 infection between June 2021 and July 2021 was included in the study. Patients with polymerase chain reaction (PCR) positive with oxygen saturation less than 90% were included in the study. Patients who were admitted directly to the intensive care unit or those who needed mechanical ventilation at admission were excluded from the study. Patients with chronic liver disease and chronic gastritis were also excluded from the study. All subjects gave their informed consent for inclusion before they participated in the study. Ethical board approval was taken before the start of the study from Peoples University of Medical and Health Sciences For Women (IRB Number: PUMHSW/IRB/COVID-19/14).

Four hundred and twelve (412) participants were enrolled in the study via consecutive convenient non-probability sampling. Participants’ symptoms and demographics were noted in a self-structured questionnaire. They were asked about GI symptoms such as anorexia, diarrhea, nausea, vomiting, abdominal pain, and others on the first day of admission, before the start of medication. Those with a history of hepatitis and chronic liver disease were excluded from the study.

The collected data were analyzed using Statistical Package for Social Sciences (SPSS), version 23.0 (IBM Corp., Armonk, NY). Mean and standard deviation (SD) were calculated for numerical data, while we calculated frequencies and percentages for categorical data. Frequencies were compared using the chi-square test. A p-value of less than 0.05 meant that there is a significant difference in the value between the two groups and the null hypothesis is void.

## Results

Out of the total 412 patients, 222 (53.8%) participants were male. The mean age at the time of enrollment was 42 ± 6 (Table 1).

**Table 1:** Characteristics of participants at the time of enrollment BPM: breaths per minute, CRP: C-reactive protein, IU: international unit, LDH: lactate dehydrogenase, mg/L: milligrams per liter, SD: Standard deviation

Characteristics at time of enrollment	Mean ± SD (n=412)
Age (in years)	42 ± 6
Male (%)	222 (53.8%)
Respiratory rate (BPM)	30.1 ± 4.8
CRP (mg/L)	131.7 ± 22.2
LDH (IU)	314.2 ± 87.2
Oxygen saturation (%)	88.1 ± 3.2

Approximately 261 (63.3%) participants had a minimum of one GI symptom. The most common symptom was anorexia (43.9%), followed by diarrhea (24.7%) and nausea/vomiting (17.9%) (Table 2).

**Table 2:** Frequency of gastrointestinal symptoms in study participants

Symptoms	Frequency (%)
Anorexia	181 (43.9%)
Diarrhea	102 (24.7%)
Nausea & vomiting	74 (17.9%)
Abdominal pain	51 (12.3%)
Bloating	14 (3.3%)
Heartburn	12 (2.9%)

## Discussion

Our study demonstrated that 63.3% of the participants showed at least one GI symptom, in which anorexia (43.9%) was the most frequent, followed by diarrhea (24.7%) and nausea/vomiting (17.9%). In concordance with the results of our study, a comprehensive study conducted in 2020 reported the frequency of the symptoms, with anorexia (26.1%) being the most common symptom, followed by diarrhea (13.5%) and nausea/vomiting (9.4%) [11]. Summarised reports from Wuhan, published in 2020, stated that approximately 79% of the patients reported complaints of GI manifestations, including diarrhea, anorexia, nausea, vomiting, abdominal pain, and GI bleeding [12]. Another similar study, conducted in 2020, in China including a large sample size examined COVID-19 patients with and without the prevalence of GI symptoms. The study showed that anorexia (39.9%-50.2%) was the most common symptom experienced by adults, followed by diarrhea (2%-49.5%) in both adults and children, and vomiting was more frequently observed in children [7]. In a large systematic review and meta-analysis of 47 studies conducted in 2020, that included 10,890 unique patients, GI symptoms (i.e., nausea, vomiting, abdominal pain, and diarrhea) were observed in less than 10% of patients with COVID-19, and abnormal liver enzymes and tests (aspartate aminotransferase (AST), alanine aminotransferase (ALT), and bilirubin) were observed in approximately 15%-20% of patients with COVID-19 [13]. As compared to adults, GI manifestations are more commonly reported among children [14]. However, the trends in the prevalence of GI symptoms are seen to increase with the progression of the pandemic. This could potentially be due to the fact that the virus is constantly mutating; one of its most recent forms is the delta variant. It is suggested that this variant has been reported to significantly cause more GI manifestations [15].

The detection of SARS-CoV-2 nucleic acids in the stool samples suggests its ability to infect the GI tract [14]. Literature has explained the potential cause of the association between COVID-19 and GI manifestations. In order for SARS-CoV-2 to enter into the host cell, the angiotensin-converting enzyme 2 (ACE2) receptor plays a major role. SARS-CoV-2 attacks the GI tract by combining with ACE2 receptors located in the glandular cells of gastric, duodenal, and rectal epithelial cells, as well as in enterocytes of the small intestine [16]. Additionally, once SARS-CoV-2 has invaded the digestive tract, the link between intestinal microbiota and pro-inflammatory cytokines could potentially invade the GI tract [17]. Another possible explanation is that COVID-19 patients are generally treated with antibiotics (abidol, ribavirin) and nonsteroidal anti-inflammatory drugs (NSAIDs); therefore, drug-induced GI manifestations should be differentiated from the ones caused by the virus itself [18].

Our study points towards the issue that COVID-19 patients who only show GI symptoms might not be given the needed attention. This is due to the fact that other common symptoms are given more importance like fever, cough, shortness of breath, etc. Therefore, awareness should be spread regarding the association between COVID-19 and the digestive tract. This would help in tracking the disease with proper management to help control its transmission. However, this was a single-center study; therefore, we suggest that further studies should be conducted in multiple centers with a larger sample size in order to include a diverse data set and to confirm the results of our study.

## Conclusions

Our study indicates a high frequency of COVID-19 patients reporting GI symptoms. Anorexia, diarrhea, nausea, and vomiting were commonly reported symptoms. Therefore, COVID 19 testing should be considered in patients presenting with GI symptoms, particularly in hospitalized patients with the new onset of GI symptoms, outpatients with the new onset of GI symptoms for over 48 hours, and in patients with established GI disease. Additionally, new guidelines should also consider including management of GI symptoms in COVID-19.

## References

[1] Silva FA, Brito BB, Santos ML (2020). COVID-19 gastrointestinal manifestations: a systematic review. Rev Soc Bras Med Trop.

[2] Gorbalenya AE, Baker SC, Baric RS (2020). The species severe acute respiratory syndrome-related coronavirus: classifying 2019-nCoV and naming it SARS-CoV-2. Nat Microbiol.

[3] Sharma A, Tiwari S, Deb MK, Marty JL (2020). Severe acute respiratory syndrome coronavirus-2 (SARS-CoV-2): a global pandemic and treatment strategies. Int J Antimicrob Agents.

[4] Wiersinga WJ, Rhodes A, Cheng AC, Peacock SJ, Prescott HC (2020). Pathophysiology, transmission, diagnosis, and treatment of coronavirus disease 2019 (COVID-19): a review. JAMA.

[5] (2021). Signs of coronavirus (COVID-19). https://www.webmd.com/lung/covid-19-symptoms#1.

[6] Huang C, Wang Y, Li X (2020). Clinical features of patients infected with 2019 novel coronavirus in Wuhan, China. Lancet.

[7] Tian Y, Rong L, Nian W, He Y (2020). Review article: gastrointestinal features in COVID-19 and the possibility of faecal transmission. Aliment Pharmacol Ther.

[8] Lin L, Jiang X, Zhang Z (2020). Gastrointestinal symptoms of 95 cases with SARS-CoV-2 infection. Gut.

[9] Rokkas T (2020). Gastrointestinal involvement in COVID-19: a systematic review and meta-analysis. Ann Gastroenterol.

[10] Gul F, Lo KB, Peterson J, McCullough PA, Goyal A, Rangaswami J (2020). Meta-analysis of outcomes of patients with COVID-19 infection with versus without gastrointestinal symptoms. Proc (Bayl Univ Med Cent).

[11] Zhao Y, Cao Y, Wang S, Cai K, Xu K (2020). COVID-19 and gastrointestinal symptoms. Br J Surg.

[12] Dan F, Jingdong MA, Jialun G, Muru W, Yang S, Dean T, Peiyuan LI (2020). Manifestations of digestive system in hospitalized patients with novel coronavirus pneumonia in Wuhan, China: a single-center, descriptive study. Chin J Dig.

[13] Sultan S, Altayar O, Siddique SM (2020). AGA institute rapid review of the gastrointestinal and liver manifestations of COVID-19, meta-analysis of international data, and recommendations for the consultative management of patients with COVID-19. Gastroenterology.

[14] Jahangir M, Nawaz M, Nanjiani D, Siddiqui MS (2021). Clinical manifestations and outcomes of COVID-19 in the paediatric population: a systematic review. Hong Kong Med J.

[15] (2021). The COVID-19 delta variant: everything we know about it so far, according to experts. https://www.health.com/condition/infectious-diseases/coronavirus/delta-variant-covid-19.

[16] Xiao F, Tang M, Zheng X, Liu Y, Li X, Shan H (2020). Evidence for gastrointestinal infection of SARS-CoV-2. Gastroenterology.

[17] Zhang D, Li S, Wang N, Tan HY, Zhang Z, Feng Y (2020). The cross-talk between gut microbiota and lungs in common lung diseases. Front Microbiol.

[18] Han C, Duan C, Zhang S (2020). Digestive symptoms in COVID-19 patients with mild disease severity: clinical presentation, stool viral rna testing, and outcomes. Am J Gastroenterol.

